# Dependency of Anion and Chain Length of Imidazolium Based Ionic Liquid on Micellization of the Block Copolymer F127 in Aqueous Solution: An Experimental Deep Insight

**DOI:** 10.3390/polym9070285

**Published:** 2017-07-19

**Authors:** Jignesh Lunagariya, Nadavala Siva Kumar, Mohammad Asif, Abhishek Dhar, Rohit L. Vekariya

**Affiliations:** 1 Department of Chemistry, College of Chemistry and Materials Science, Jinan University, Guangzhou 510632, China; jignesh.lunagariya@gmail.com; 2 Department of Chemical Engineering, King Saud University, P.O. Box 800, Riyadh 11421, Saudi Arabia; masif@ksu.edu.sa; 3 Department of Science & Technology, Government of West Bengal, 26/B, Block-DD, Sector-I, Salt Lake, Kolkata 700064, India; abhidhar2000@gmail.com; 4 Department for Management of Science and Technology Development, Ton Duc Thang University, Ho Chi Minh City, Vietnam; 5 Faculty of Applied Sciences, Ton Duc Thang University, Ho Chi Minh City, Vietnam

**Keywords:** micellization, surface activity, block copolymers, ionic liquids, DLS, viscosity

## Abstract

The non-ionic triblock copolymer, Pluronic^®^ F127, has been selected to observe its interaction with ionic liquids (ILs) in aqueous solutions by using DLS, surface tension, and viscosity measurements. The Critical Micelle Concentration (CMC) of F127 increased with the addition of ILs, which appeared logical since it increases the solubility of PPO (and PEO) moiety, making it behaves more like a hydrophilic block copolymer that is micellized at a higher copolymer concentration. The results from DLS data showed good agreement with those obtained from the surface tension measurements. Upon the addition of ILs, the tendency in micellar size reduction was demonstrated by viscosity results, and therefore, intrinsic viscosity decreased compared to pure F127 in aqueous solution. The results were discussed as a function of alkyl chain length and anions of imidazolium based ILs.

## 1. Introduction

Interaction in aqueous solutions between Poly(ethylene oxide)–poly(propylene oxide)–poly(ethylene oxide), (PEO–PPO–PEO) like water soluble block copolymers and surfactants is very crucial in laundry detergents cosmetics, paint, coating, emulsification, and petroleum industries along with pharmaceutical interest [1,2]. Of late, the area of their functions is much wider covering their uses in many walks such as gene delivery [3], drug delivery [4], nanoparticles synthesis [5], and as templating agents [6]. A large number of these applications are concerned with the formation of micelles and hence, micellization of block copolymer has generated substantial interest in the literature [7]. The interaction along with the aggregation behavior of mixed surfactants in solution and at interfaces has been rigorously investigated using various techniques such as small angle neutron scattering (SANS), dynamic light scattering (DLS), fluorescence, surface tension, viscosity, conductivity, and cyclic voltammeter [8,9,10,11,12]. As Pluronics^®^ are often employed in complex environments, for instance, in presence of other additives or amphiphile, studies on their effect on solution behavior of copolymers can help to optimize properties based on performance for various applications. Additives such as electrolytes [13] and ionic liquids (ILs) [14,15]; non-electrolytes including urea [16], alkanols [17], and inorganic salts [18,19]; and surfactants [20] have been studied for their effect on the aggregation behavior of triblock copolymers. Organic additives have also been investigated for their effect in different systems [21,22]. The effect of alcohol is dependent upon its capability to reside in different micro domains and ability to alter the interfacial curvature by swelling different block copolymers. Phase behavior and performance of surfactants have been affected by the swelling tune of different co-solvents [23]. The addition of ethanol, for example, weakens the block segregation due to better solvency as compared to that of water [24].

Due to the electrochemical window and wide liquid temperature range, ILs show high extractive selectivity and inflammability with negligible volatility and high thermal stability. As a result, ILs plays a pivotal role as alternative media in wide-spread areas [25,26,27,28]. ILs is typically made of large molecular cations and anions and henceforth, several combinations between them are feasible. This factor means ILs are often termed as ‘designer solvent’ or considered as ‘task-specific IL’. The role of ILs on aggregation behavior of the surfactants/block copolymers has been studied and it could be beneficial to elevate the properties of surfactants/block copolymers and that basically widens its application in related industrial areas. The pyridinium and imidazolium based ILs is considered to be an important class of cations in the surface active ILs family, and their aggregation has been thoroughly studied in aqueous solutions [29,30,31,32,33]. Recently, P. Venkatesu and his co-worker has shown the outcome of 1-alkyl-3-methylimidazolium chloride based ILs on the thermo-responsive triblock copolymer, (PEG-PPG-PEG) by using various methods such as fluorescence spectroscopy, viscosity, dynamic light scattering, and Fourier transform infrared spectroscopy [34]. Thus, we have focused our present work to establish a correlation between variations of imidazolium based ILs possessing different chain lengths and anions on the micellization of Pluronic^®^ F127 in aqueous media. Critical micelle temperatures, micellization, and cloud points are greatly affected by the addition of salts as well conventional surfactants of both cationic and anionic nature [35].

In the present study, we have reported the effect of ILs containing different chain lengths and anions on Micellization behavior of Pluronic^®^ F127 block copolymer in aqueous media. The DLS study has been utilized to evaluate the size of F127 micelles in the presence of imidazolium based ILs of different chain lengths and containing different anions, which is further assisted by surface tension and viscosity measurements. A thorough study of micellization of F127 with different ILs and their concentrations in aqueous solution has been carried out.

## 2. Materials and Methods

**Scheme 1:**
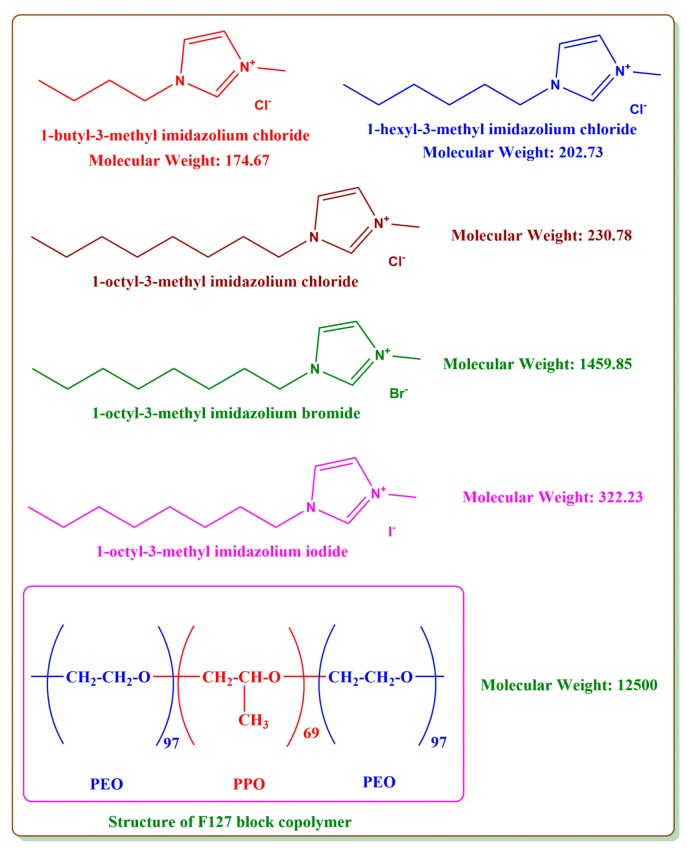
Chemical structures of synthesized ILs and Pluronic block copolymer F127 along with molecular weight.

Pluronic^®^ F127 (EO_97_PO_69_EO_97_), possessing molecular weight 12,500 g·mol^−1^, was obtained from Sigma-Aldrich (Sigma-Aldrich, Shanghai, China). Ionic liquids (ILs) were synthesized using standard procedure reported in literatures [29,36,37]. For their characterization, ^1^H NMR, TGA, and IR methods were employed. The water content of ILs, measured by Karl Fisher analysis (HB-WS Coulometric Karl Fischer Moisture Tester, Chongqing, China), was always less than 0.05%. Their storage was maintained at 60 °C in vacuum. The chemical structures of synthesized ILs and Pluronic^®^ block copolymer F127 along with molecular weight are given in Scheme 1. Aqueous solutions of ionic liquids and Pluronic^®^ F127 were prepared using appropriate amounts of degassed Millipore grade distilled water.

### 2.1. Dynamic Light Scattering (DLS)

To determine the apparent hydrodynamic radius of F127 micelles in the presence and absence of ILs in aqueous solution, Dynamic Light Scattering (Malvern, Worcestershire, UK) was used. These measurements were performed at 90° scattering angle on solutions using a Malvern 4800. Autosizer equipped with a 192 channel digital correlator 7132 and coherent (Innova) Ar-ion laser at a wavelength of 514.5 nm. The detailed of theoretical approach of DLS analysis is given in the supplementary section. The apparent equivalent hydrodynamic radius (*R*_h_) of the micelles was computed with the help of the following Stokes–Einstein Equation (1).

(1) $$R_{h}=\frac{K_{B}T}{6\pi \eta D_{0}}$$

where *D*_0_ is the diffusion coefficient, *K*_B_ is the Boltzmann constant, and η is the viscosity of solvent, i.e., water at temperature *T*.

#### Theoretical Approach for DLS Analysis

The normalized intensity autocorrelation function, *g*^(2)^(τ), is given as

(2) $$g^{\left(2\right)}\left(\tau \right)=\frac{\langle I\left(0\right)I\left(\tau \right)\rangle }{{\langle I\rangle }^{2}}$$

where 〈I〉 is the ensemble averaged intensity. The above functions is related to *g*^(1)^(τ), which is the first order correlation function, as follows (Siegert relationship),

(3) $$g^{\left(2\right)}\left(\tau \right)=\beta +A{\left|g^{\left(1\right)}\left(\tau \right)\right|}^{2}$$

where, the parameter *A* depends upon the geometry of scattering surface and is independent of τ while the parameter β is the base line.

To compute the apparent diffusion coefficient of the micelles (*D*_a_), the following relationship is used,

(4) $$ln\left[g^{\left(1\right)}\left(\tau \right)\right]=ln\beta -\bar{\Gamma }\tau +\frac{\mu_{2}\tau^{2}}{2}$$

where µ_2_ is the variance in the relaxation rate distribution and $\bar{\Gamma }$ is the mean relaxation rate. The average decay rate can be evaluated from the time dependence of the electrical field autocorrelation function *g*^(1)^(τ) using a modified cumulants method.

### 2.2. Surface Tension (ST)

We used a surface tensiometer (Data physics, Germany, Model DCAT 11) for the measurement of the surface tension of both the pure system as well as their mixtures. The experiments were carried out with an uncertainty of ±0.03 m·Nm^−1^ using Wilhemy plate method at a constant temperature of 30 ± 1 °C. For the purpose of calibration, the surface tension of double distilled water (71.3 ± 0.2 m·Nm^−1^) was used. The surface tension measurement was carried out by the successive addition of the stock solution in pure double distilled water. Three replicate measurements were always made to ensure the accuracy of data. The estimated data reproducibility of surface tension measurement was within ±0.1 × 10^−5^ M.

### 2.3. Viscosity

We have used Ubbelohde type (suspended level capillary) viscometer for the measurement of viscosity at 30 ± 0.1 °C. Each measurement was carried using a clean and dried viscometer. Three replicate runs were always made to ensure the accuracy of measurements. We recorded the time of flow of a constant volume of solution through the capillary using a calibrated stopwatch. Since the flow time always exceeded 170 s, no kinetic corrections were made.

## 3. Results and Discussion

### 3.1. Dynamic Light Scattering (DLS) Measurements

**Figure 1:**
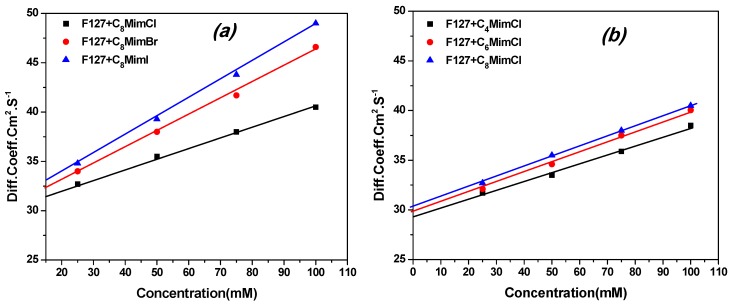
DLS curves for F127 micelles in presence of IL (**a**) effect of anions and (**b**) effect of alkyl chain length on micellization.

DLS measurements were performed on 5% (*w*/*v*) F127 solutions in water by varying the concentration of ionic liquids (ILs) at 30 °C. The effect of chain lengths and anions of ILs were investigated by measuring the diffusion coefficient of the copolymers aggregates to examine the change in the size of micelles. The results are presented in Figure 1. The increase in diffusion coefficient with the increase of ILs concentration can be attributed to the decreases in the sizes of micelles.

The experimental data was analyzed using the constrained regularization method (CONTIN). It reveals a unimodel distribution of relaxation rate, thus highlighting the applicability of cumulative results. The average value of *τ* derived from cumulative analysis was used to compute the diffusion coefficient as well as hydrodynamic radius, *R*_h_, using Stokes–Einstein equation (Equation (1)). The apparent radii of the F127 micelles with various cationic ILs as derived by CONTIN analysis of the DLS data are summarized in Table 1. For pure 5% (*w*/*v*) of F127 aqueous solution in absence of IL, *R*_h_ is 13.42 nm at 30 °C, which is in good agreement with reported value of 12.18 nm for pure 1% (*w*/*v*) F127 aqueous solution [38]. Upon addition of ILs, the polydispersity of micelles enhances, which were observed together with the decrease in the size of F127 micelles (Table 1). This implies that the micelle proceeds via reduction in size controlling spherical geometry, which is also confirmed from the DLS data. An imidazolium IL with a longer alkyl chain (i.e., 8 carbon) and iodide as anion has a deep-rooted effect on the quenching of size of micelles as compared to lower carbon chains as well as other anions.

### 3.2. Surface Tension Measurements

**Figure 2:**
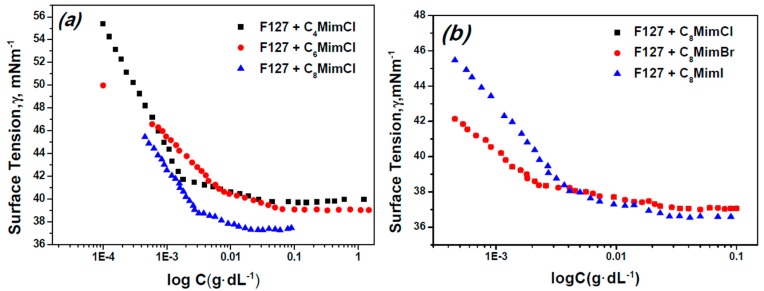
Surface tension behavior of F127 in presence of various IL. (**a**) Effect of chain length and (**b**) effect of anions.

The effect of concentration and ILs on the surface tension (γ) at 30 °C for the aqueous solution of Pluronic^®^ F127 is shown in Figure 2. The influence of both alkyl chain lengths and anions of ILs was investigated here.

Unlike conventional non-ionic surfactants where a sharp inflection point is usually noticed, the present γ vs. log C plots, shown in Figure 2, exhibit different behavior. The surface tension isotherms show two break points over the concentration range investigated here, thus dividing the curve in three regions. At low concentrations, the surface tension shows a strong linear dependence on the concentration. At intermediate concentrations, the concentration dependence of the surface tension is much weaker. On the other hand, surface tension is independent of the concentration at high concentrations as the curves tend to be nearly flat [39]. Similar behavior has been reported earlier for PEO/PPO/PEO copolymer solutions in water and in the presence of added salt [40,41]. The constancy of the surface tension value can be attributed to the formation of micelle like structures. When the surface tension becomes almost independent of the concentration is defined as the critical micelle concentration (CMC). At this concentration of the surfactant, micelles begin to appear and any further addition of the surfactant goes to micelles’ formation. In fact, CMC corresponds to a maximum change in the gradient of solution property with respect to the concentration. The experimental results are presented in Table 2. The CMC value of Pluronic^®^ F127, listed in Table 2, is in good agreement with the reported literature [42].

In the presence of ILs, a second break point occurs at a higher concentration, thereby leading to an increase in the CMC values. This is caused by the increase in the solubility of PPO moiety (and PEO), thus behaving like a hydrophilic block copolymer that is micellized at high copolymer concentration. γ_cmc_ represents the surface tension that corresponds to CMC of pure copolymer and mixed systems. As shown in Table 2, the value of γ_cmc_ decreases upon the addition of ILs indicating that mixed systems are more surface active than pure copolymer. Moreover, the less negative $∆G_{\mathrm{mic}}$ values (calculated using Equation (5)) are confirming thermodynamically favorable de-micellization of F127 micelles in presence of diverse cationic ILs.

(5) $$∆G_{\mathrm{mic}}=RTlnX_{\mathrm{cmc}}$$

where, *R* is the gas constant (8.314 KJ/mole), *T* is temperature (303.15 K), and *X*_cmc_ is the mole fraction of the surfactant at CMC. In aqueous solutions when CMC is below 10^−1^ M, Equation (5) can be written as [43]

(6) ∆Gmic=RTln(CMCw)

where, *w* is water molarity. The calculated $∆G_{\mathrm{mic}}$ for F127 in presence of diverse cationic ILs are provided in last column of Table 2.

### 3.3. Viscosity Measurements

**Figure 3:**
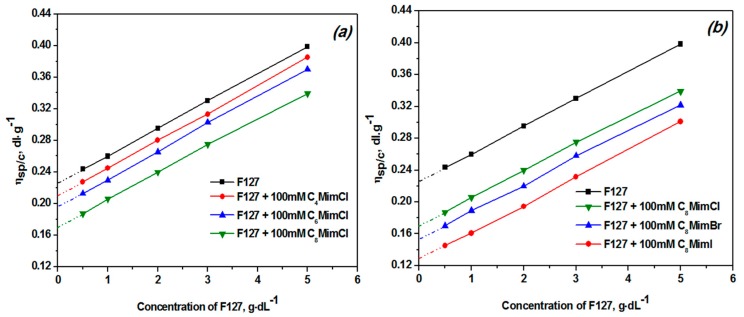
Concentration dependence of the reduced viscosity of F127 in presence of 100 mM ILs at 30 °C; (**a**) influence of chain lengths, (**b**) influence of anion.

The intrinsic viscosity [η] of F127 in water and 100 mM ILs solutions at 30 °C are presented in Figure 3 and Table 3. The reduced viscosities are derived from {(η_sp_ = η_r_ − 1, (η_r_ = η/η_0_))}, where *η*_0_and η represent viscosities of the solvent and the copolymer solution, respectively}. In all the cases, the plots are linear, which is a typical feature of an uncharged polymeric behavior. These plots can be used to obtain intrinsic viscosity [η] required for determining the Huggins constant. However, intrinsic viscosity varied with the alteration in alkyl chain length (C_4_, C_6_, C_8_) and/or anions (Cl−, Br−, I−) of ILs. The reduction in intrinsic viscosities can be attributed to the dehydration of PEO moiety resulting more compact structures. Interactions between F127 micelles and imidazolium based ILs can be found from the viscosity behavior of F127 solutions. The decrease in the reduced viscosity in presence of cationic ILs demonstrates the micellar size reduction, which is also supported by DLS results.

The viscosity of F127 solution was used to compute the intrinsic viscosity [η] and Huggin’s constant, *K*_H_, (Table 3) with the help of the following relationship,

(7) $$\eta_{\mathrm{Sp}/C}=\left[\eta \right]+{\left[\eta \right]}^{2}K_{H}C$$

where, $\eta_{\mathrm{Sp}/C}$ is the reduced viscosity. The high intrinsic viscosity for pure F127 is due to the more extended conformation of the molecules.

**Scheme 2:**
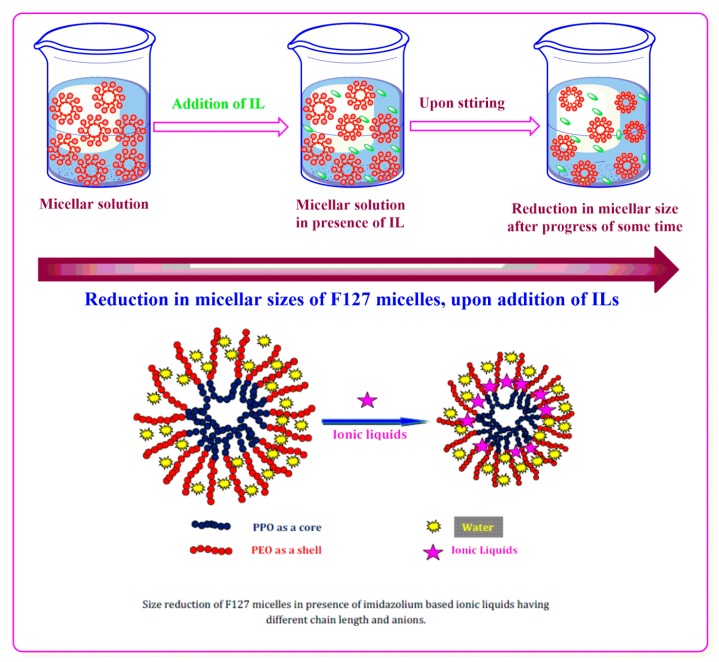
Micellization process and micellar size reduction due to ILs.

The increase in viscosity may also be caused by aggregation of copolymers into micelles with increasing copolymer concentration. Adding ILs composed of various cationic head groups leads to a reduction in [η] and an enhancement in *K*_H_ as compared to corresponding values of water. The decrease in [η] exhibits the de-micellization caused by micellar hydration and improved solvent quality of the mixed water + ILs system, leading to a size reduction of micelles of spherical geometry. Similar observations have earlier been reported for diverse Pluronics^®^ block copolymers in the presence of additives [44]. In fact, the increase in the *K*_H_ values due to the addition of ILs indicates a substantial strengthening of the attractive interactions between solvent (water + ILs) and the hydrophilic part of the block copolymer molecules due to hydration effects. It is noteworthy that the viscosity data also supports the results obtained from the DLS measurements. Upon addition of ILs to aqueous solution of block copolymer micelles and how ILs influenced on micelles to reduced size is given in Scheme 2.

## 4. Conclusions

This study investigated the role of alkyl chain and anions of imidazolium based ILs having different chain length and anions such as C_4_–C_8_ and Cl−, Br− and I− respectively, for F127 micelles. The DLS data yields information regarding the evolution of the micellar form by altering the nature of cationic head group of ionic liquids when added to F127 aqueous solution. The DLS data is supported well by the results observed from surface tension measurements. The highest degree of de-micellization was observed in the case of C_8_MimI. The addition of ILs significantly affected the solution properties which were reflected in both the solution viscosity and the micellar size. The viscosity data display the propensity in the reduction of micellar size due to the addition of ILs and hence, intrinsic viscosity drops off as compared to pure F127 aqueous solution. With the addition of ILs to micellar solution, a linear increase in CMC, shifting of micellization and micelle growths to higher concentration were noticed. In a nutshell, upon addition of ionic additive (ILs), due to hydration of this ionic part at PEO-PPO interface; micellization becomes less facile in block copolymers, which is supported by Gibbs free energy, i.e., negative magnitude of $∆G_{\mathrm{mic}}$ decreases upon addition of ILs as an additive and this value confirms the lesser thermodynamic feasibility of the process.

## Figures and Tables

**Table 1:** Parameters obtained from DLS measurements.

System	*D*_0_ × 10^8^ cm^2^ s^−1^	*R*_h_ (nm)	*K*_D_ (Lit.mole^−1^)	*R*^2^
F127 in water	23.62	13.42	35.68	94.90
5% *w*/*v* F127 + C_8_MimCl	32.1	9.03	3.10	98.57
5% *w*/*v* F127 + C_4_MimCl	29.2	10.85	3.12	99.35
5% *w*/*v* F127 + C_6_MimCl	29.7	10.67	3.51	98.41
5% *w*/*v* F127 + C_8_MimBr	30.2	10.49	3.43	99.31
5% *w*/*v* F127 + C_8_MimI	29.45	10.76	6.38	98.36

**Table 2:** CMC values parameters obtained from surface tension measurements.

System	CMC (g·dL^−1^)	γ_cmc_ (m·Nm^−1^)	Δ*G*_mic_ (KJ/mol)
Pure F127	0.02950	41.12	−19.00
F127 + C_4_MimCl	0.03400	37.74	−18.65
F127 + C_6_MimCl	0.04300	38.06	−18.05
F127 + C_8_MimCl	0.03668	37.05	−18.65
F127 + C_8_MimBr	0.02100	37.31	−19.01
F127 + C_8_MimI	0.03180	36.65	−18.3

**Table 3:** Effect of ILs on the intrinsic viscosity [η] and Huggins constant, *K*_H_, for F127 at 30 °C.

Solution Systems	[η] (dL·g^−1^)	*K*_H_
F127	0.221	0.64
F127 + C_4_MimCl	0.201	0.75
F127 + C_6_MimCl	0.189	0.89
F127 + C_8_MimCl	0.165	1.10
F127 + C_8_MimBr	0.150	1.24
F127 + C_8_MimI	0.121	2.08
